# Cost-effectiveness of ablation of ventricular tachycardia in ischaemic cardiomyopathy: limitations in the trial evidence base

**DOI:** 10.1136/openhrt-2019-001155

**Published:** 2020-01-28

**Authors:** Yang Chen, Manuel Gomes, Jason V Garcia, Ross J Hunter, Anthony W Chow, Mehul Dhinoja, Richard J Schilling, Martin Lowe, Pier D Lambiase

**Affiliations:** 1 Cardiology, Barts Health NHS Trust, London, UK; 2 Institute of Cardiovascular Science, University College London, London, UK; 3 Department of Applied Health Research, University College London, London, UK

**Keywords:** VT ablation, Markov model, incremental cost-effectiveness ratio

## Abstract

**Objective:**

Catheter ablation is an important treatment for ventricular tachycardia (VT) that reduces the frequency of episodes of VT. We sought to evaluate the cost-effectiveness of catheter ablation versus antiarrhythmic drug (AAD) therapy.

**Methods:**

A decision-analytic Markov model was used to calculate the costs and health outcomes of catheter ablation or AAD treatment of VT for a hypothetical cohort of patients with ischaemic cardiomyopathy and an implantable cardioverter-defibrillator. The health states and input parameters of the model were informed by patient-reported health-related quality of life (HRQL) data using randomised clinical trial (RCT)-level evidence wherever possible. Costs were calculated from a 2018 UK perspective.

**Results:**

Catheter ablation versus AAD therapy had an incremental cost-effectiveness ratio (ICER) of £144 150 (€161 448) per quality-adjusted life-year gained, over a 5-year time horizon. This ICER was driven by small differences in patient-reported HRQL between AAD therapy and catheter ablation. However, only three of six RCTs had measured patient-reported HRQL, and when this was done, it was assessed infrequently. Using probabilistic sensitivity analyses, the likelihood of catheter ablation being cost-effective was only 11%, assuming a willingness-to-pay threshold of £30 000 used by the UK’s National Institute for Health and Care Excellence.

**Conclusion:**

Catheter ablation of VT is unlikely to be cost-effective compared with AAD therapy based on the current randomised trial evidence. However, better designed studies incorporating detailed and more frequent quality of life assessments are needed to provide more robust and informed cost-effectiveness analyses.

**Key questions**

**What is already known about this subject?**

- There are few cost-effectiveness studies of catheter ablation of ventricular tachycardia (VT). Previous studies have used non-randomised clinical trial (RCT) data as well as expert opinion to inform their models and conclusions or have used a within-trial analysis with a short time horizon. These have generated conclusions that ablation of VT was cost-effective according to the threshold set by the National Institute for Health and Care Excellence though the brittleness of the objective data with which this was based makes such conclusions questionable.

**What does this study add?**

- We have demonstrated that among all RCT data in this area, the assessment of health-related quality of life (HRQL) within trials is poor. Given VT ablation does not confer prognostic benefit, the main determinant of it cost-effectiveness will be the additional monetary costs of the treatment and the quality of life gained. Because of the paucity of patient-reported HRQL, the difference in quality of life was marginal. This was the dominant force in determining the incremental cost-effectiveness ratio and in our particular model, resulted in the conclusion that VT ablation was unlikely to be cost-effective.

**How might this impact on clinical practice?**

- By using a well-studied condition such as VT as an exemplar to highlight the brittleness of HRQL data in cardiology RCTs, our message is that more robust assessment of patient-reported HRQL should be the standard, particularly in trials involving expensive or widely used treatments. Patients need to be at the heart of decision-making, advised by clinical expertise. Moving forward, health economic research should place more emphasis on patient-reported outcome measures when creating the evidence base to inform funding decisions, quality of life assessments and cost-effectiveness analyses.

## Introduction

Catheter ablation is an important treatment for ventricular tachycardia (VT). Six randomised clinical trials (RCT)^1–6^ have examined the role of VT ablation in patients with ischaemic cardiomyopathy. A meta-analysis concluded that ablation reduced VT events, implantable cardioverter-defibrillator (ICD) therapies and readmissions, but had no effect on mortality.^7^ Consequently, European and American guidelines offer similar recommendations for the role of catheter ablation. It is considered a first-line treatment only in recurrent cases of VT despite antiarrhythmic drugs (AAD) or in those who are intolerant of AADs.^8 9^

Although VT has multiple different aetiological factors, the increasing number of patients with ischaemic cardiomyopathy means that it is reasonable to project a growing number of VT ablations in the future. Catheter ablation can be a time-intensive and difficult procedure and thus a focus on its comparative effectiveness and specifically its cost-effectiveness is of increasing importance, particularly to maximise the efficiency of resource use in a time when rising costs are a problem for all healthcare systems.

The current evidence base for the cost-effectiveness of VT ablation is limited—one analysis was conducted before the publication of RCTs and one other study was based on only a single RCT.^10 11^

By synthesising all available RCT evidence, our study uses more data, and from a broader cohort of patients, to address whether VT ablation is a cost-effective treatment for individuals with ischaemic cardiomyopathy implanted with an ICD.

## Background

Cost-effectiveness analyses (CEA) are usually expressed using the incremental cost-effectiveness ratio (ICER). The term CEA is also often used interchangeably with cost-utility analysis, particularly in a non-specialist setting. This is to convey the idea that there requires calculation of monetary costs and the *preference-weighted health utilities* (*ie, benefits*) for patients of the treatments being compared.

*The ICER represents the additional monetary cost incurred for a given treatment of interest, compared with another, in order to gain one additional quality-adjusted life-year* (*QALY*).^12^ Here, the definition of one QALY is equal to 1 year spent in perfect health. QALYs are calculated through collection of health-related quality of life (HRQL) data.

A treatment is judged to be cost-effective if the associated ICER is below the societal willingness-to-pay (WTP) threshold. For the UK’s National Health Service (NHS), the WTP is set by the National Institute for Health and Care Excellence (NICE), and is between £20 000 and £30 000 per QALY.^13^

## Methods

### ​Overview

In order to determine whether VT ablation is cost-effective, a health economic model was built to forecast events beyond those limited to the RCT follow-up. To account for recurrent clinical events, a Markov model instead of a decision tree was employed to simulate a hypothetical cohort of 1000 patients undergoing a VT ablation strategy and 1000 patients undergoing an AAD strategy. Following previous modelling studies in catheter ablation,^10 14^ we considered a 5-year time horizon for the base-case analysis, striking a balance between clinical relevance and available evidence on long-term cost and outcomes in this population. Scenarios with both a 10-year and lifetime horizon were considered in sensitivity analyses. Costs were considered from a single UK NHS hospital perspective, and calculated using 2017/2018 NHS reference tariffs. Discounting was applied to costs and utilities, to account for time preference. This was standardised to NICE’s recommended annual rate of 3.5%.^13^ The decision-analytic model was programmed and analysed in Microsoft Excel V.2013 (Microsoft, Redmond, Washington, USA).

### ​Model structure

**Figure 1:**
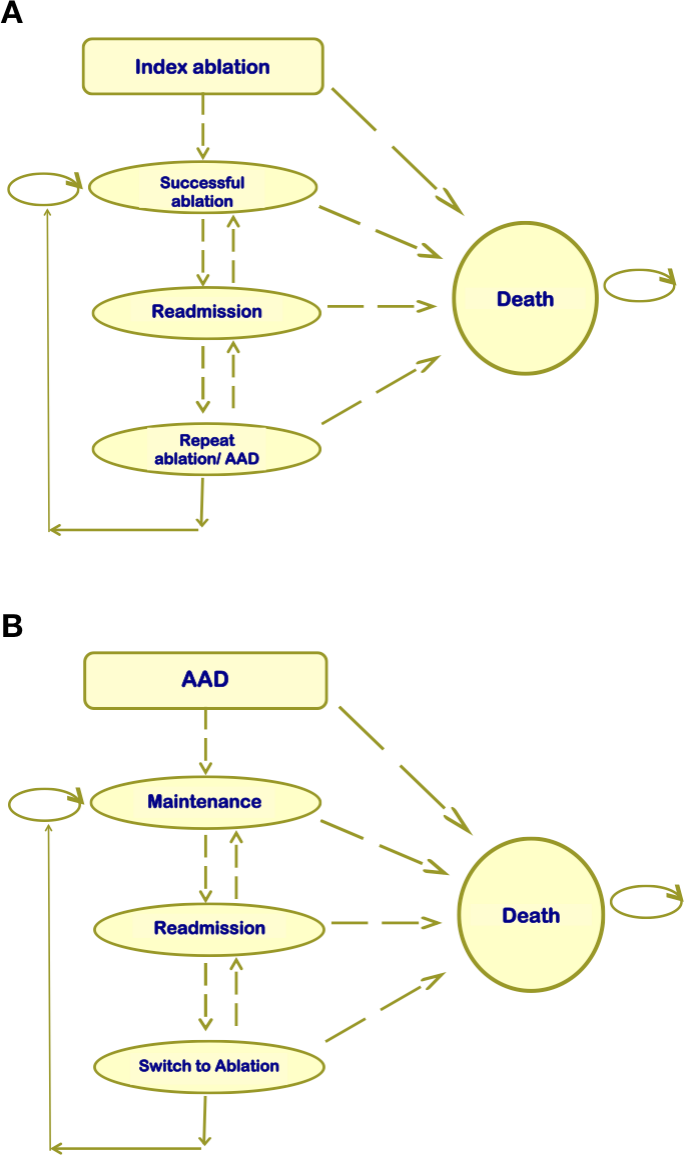
Schematic of model structure used in simulation. (A) Represents the model for the ablation arm. (B) Represents the model for the AAD arm. AAD, antiarrhythmic drug.

The different health states chosen for our model were based on relevant and pragmatically measurable outcomes for each state. In the ablation arm, patients were in one of five mutually exclusive health states: *death, successful ablation, successful ablation with adverse event, repeat ablation* and *readmission*. Patients in the AAD group were in one of the five mutually exclusive health states: *death, AAD maintenance, AAD maintenance with adverse event, readmission* and *switch to ablation* (see figure 1). Readmission rates were derived according to reported hospitalisations, VT storms or repeated ablations from the RCTs.

A cycle length of 1 month (giving a simulation of 60 cycles in the base-case scenario) was selected to adequately model the frequency of clinically relevant events, such as readmission or death. A summary of the relevant RCT data used to inform input parameters for the model is found in table 1. All patients were assumed to have an episode of VT at the start of the simulation, where index ablation or a decision to begin or continue with AAD treatment occurs. The model allows for crossover as well as repeat ablation to reflect real-life practice. Further details of the equations used to model transition probabilities are available in the online supplementary appendix A.

10.1136/openhrt-2019-001155.supp1 Supplementary data

**Table 1:** Summary of RCT-level source data

Author	Reddy *et al* 4	Kuck *et al* 2	Kuck *et al* 1	Al-Khatib *et al* 5	Sapp *et al* 3	Di Biase *et al* 6
Name of trial	SMASH VT	VTACH	SMS	CALYPSO	VANISH	VISTA
Sample size	128	110	111	27	259	118
Mean age	67	66	67	64	68	66
LVEF (%)	31.8	34.0	31.2	24	31.2	32.3
Proportion of patients with NYHA class III/IV	20%	NYHA IV excluded	NYHA IV excluded	14.8%	23.6% NYHA III NYHA IV excluded	34%
Control	AAD	AAD	AAD	AAD	AAD	Clinical ablation
Intervention	Ablation	Ablation	Ablation	Ablation	Ablation	Substrate ablation
Length of follow-up (months)	22	24	28	6	28	12
Mortality	11% (AAD) vs 9% (ablation)	7% (AAD) vs 10% (ablation)	19% (AAD) vs 17% (ablation)	14% (AAD) vs 15% (ablation)	28% (AAD) vs 27% (ablation)	15% (C-ablation) vs 9% (S-ablation)
Readmission (%)	19% (AAD) vs 6% (ablation)	55% (AAD) vs 33% (ablation)	44% (AAD) vs 39% (ablation)	50% (AAD) vs 38% (ablation)	31% (AAD) vs 25% (ablation)	32% (C-ablation) vs 12% (S-ablation)
Quality of life	Absent	SF-36 form at 12 and 24 months	SF-36 form at 0 and 23 months	Absent	Substudy with SF-36 form, EQ-5D, HADS, ICDC at 0, 3, 6, 12 months	Absent

AAD, antiarrhythmic drug; EQ-5D, EuroQol-5 Dimension; HADS, Hospital Anxiety and Depression Scale; ICDC, ICD Patient Concerns questionnaire; LVEF, left ventricular ejection fraction; NYHA, New York Heart Association; RCT, randomised clinical trial; SF-36, Short Form-36 questionnaire; SMS, Substrate Modification Study.

### ​Data sources

Table 2 summarises the main input variables for the model and their sources. Wherever possible, RCT-level source data were used and where data were missing, in particular related to estimating the effect size of disutilities, large registries or previously published cost-effectiveness study methodology^10 15^ was used to determine which studies to reference, with further details in online supplementary appendix B. The different components are described as follows.

**Table 2:** Model inputs for base-case analysis

Model input	AAD therapy	Ablation therapy	Distribution	Data source
Probability of death per cycle	0.839%	0.814%	Beta	Weighted average of RCTs
Initial ablation operative mortality	n/a	1%	Beta	RCTs
Pooled mean age	66	66	n/a	RCTs
Probability of transition to ‘readmission’ per cycle	1.666%	1.332%	Beta	Weighted average of RCTs
Probability of transition from ‘readmission’ to ‘repeat ablation’ per cycle	25%	19%	Beta	Large registry
Cost of initial strategy	£68	£8124	Gamma	Internal audit data, NHS and British National FormularyNF reference costs
Cost of maintenance of therapy per cycle*	£49	£10	Gamma	RCT and British National Formulary
Cost of repeat ablation/switch to ablation	£8176	£8176	Gamma	Internal audit data
Cost of readmission	£2072	£2072	Gamma	Retrospective cohort study, NHS reference costs
Utility at baseline	0.781	0.771	Beta	RCTs
Disutility of readmission	−0.02	−0.02	Beta	From review article and cohort study
Disutility of reablation	−0.04	−0.04	Beta	Registry
Disutility of reablation with adverse event	−0.13	−0.13	Beta	Registry, review article
Disutility of AAD with adverse event†	−0.06	−0.06	Beta	RCT
Discount	3.5%	3.5%	n/a	NICE

*Assume 20% of ablation arm also on amiodarone.

†Assume 1.29% rate per cycle of adverse event (AE). Costs are in UK sterling as of 2018.

AAD, antiarrhythmic drug; n/a, not applicable; NHS, National Health Service; NICE, National Institute for Health and Care Excellence; RCT, randomised clinical trial.

#### ​Clinical effectiveness

The maximum follow-up time guided by the RCT data was 28 months for clinical outcomes. Assumptions in the model including monthly mortality and readmission rates were calculated as a weighted average, accounting for the different RCT sample sizes. The mortality rate of 46% for the hypothetical cohort at 5 years is comparable with large international registry data^16^ and follow-up data from our own institution.^17^

#### ​Health-related quality of life

HRQL outcomes were taken from the VTACH, Substrate Modification Study (SMS) and VANISH studies.^1–3^ HRQL was reported using the Short Form-36 questionnaire (SF-36) across the three studies, and appropriately transformed to a utility score—in the form of an SF-6D—using a previously validated method.^18^ Although EuroQol-5 Dimension (EQ-5D) was also reported in VANISH, and is favoured by NICE, we adhered to SF-36 to allow pooling of HRQL (adjusted for RCT sample size), which was done at the group aggregate level. The effect of using EQ-5D was examined in a sensitivity analysis. The maximum follow-up time for HRQL data was 24 months. Only VANISH and SMS reported baseline HRQL—with these, the calculated utility at the beginning of the model was 0.771 for the ablation group and 0.781 for the AAD group. Details of the references for disutility values applied to adverse events and readmissions are available in online supplementary appendix B.

10.1136/openhrt-2019-001155.supp2 Supplementary data

#### ​Resource use and unit costs

Costs are reported in 2018 British sterling and also in euros, according to the latest exchange rate at the time of writing (£1=€1.12). Cost data for equipment, staffing and bed days were calculated from an institutional perspective at a large tertiary hospital in London using standard Healthcare Resource Group (HRG) codes. The cost of index and repeat ablation was calculated as a mean of 18 months’ worth of VT ablation cases (n=84), coded as either index or repeat ablation. The cost of readmission was calculated using HRG codes for bed days and staffing, and the average length of stay for VT readmissions, from registry data. Medication costs were sourced from the British National Formulary. Costs common to both treatment strategies, such as outpatient clinic follow-up, and cost of other cardiovascular medications, were not included in the analysis. The mean cost of index ablation was £8124 (€9099).

### ​Sensitivity analyses

A range of one-way sensitivity analyses were performed to assess whether the cost-effectiveness results were sensitive to plausible departures from assumptions in the base-case scenario. These deterministic sensitivity analyses included: (1) allowing for a longer time horizon—10 years and lifetime, (2) allowing for changes in baseline event rates or incremental rates in events, and (3) allowing for changes in adverse event rates associated with ablation or AAD. A probabilistic sensitivity analysis was also undertaken to characterise the overall uncertainty in the input parameters. A beta distribution was used for transition probabilities to ensure these were bounded between 0 and 1 and assigned a gamma distribution to cost parameters as these could not include negative values.

### Model set-up

Each of the 60 cycles in the model incurred a differential cost and utility function depending on the various health states that patients in the hypothetical cohort occupied. In the base-case analysis, the maximum number of QALYs per patient that was possible to accrue was 5. QALYs were calculated by summing the utility scores for each health state and transforming the data from monthly cycles to a per-year scale. The model used in this paper is available to download—see online supplementary file 2.

## Results

Our base-case scenario (table 3) suggests that catheter ablation is unlikely to be a cost-effective treatment strategy compared with AAD therapy. On average, the difference in cost between the two strategies was modest at £5657 (€6336), however the difference in QALYs was small, at only 0.039 QALYs, giving an ICER of £144 150 in 2018 UK sterling (€161 448).

**Table 3:** Base-case analysis

Strategy	Mean total cost	Mean total QALYs	Incremental cost	Incremental QALY	ICER
Ablation	£10 483 (€11 741)	2.801	£5657 (€6336)	0.039	£144 150 (€161 448)
AAD	£4826 (€5405)	2.762			

AAD, antiarrhythmic drug; ICER, incremental cost-effectiveness ratio; QALY, quality-adjusted life-year.

Additional one-way sensitivity analyses demonstrated that conclusions regarding cost-effectiveness remained unchanged in a wide range of departures from the base-case scenario (table 4). It appears that the ICER falls below the UK’s WTP threshold of £30 000 only when the mortality rate is adjusted to create a significant difference between ablation and AAD—at a monthly mortality rate of 0.839% for AAD and 0.6% for ablation—the ICER is £28 631 (€32 067).

**Table 4:** One-way sensitivity analysis and effect on ICER

Variable	Base case	Sensitivity analysis range	Incremental cost	Incremental QALY	ICER	Source of sensitivity analysis range
At end of 10 years	–	–	£4669 (€5229)	0.063	£74 469 (€84 405)	–
At lifetime horizon (until entire cohort is in death state)	–	–	£4342 (€4863)	0.063	£68 853 (€77 115)	–
Difference in HRQL at end of follow-up	0.788 (ablation) −0.769 (AAD)	0.802	£5657 (€6335)	0.007	£815 610 (€913 483)	RCT
Use of EQ-5D data from VANISH trial as HRQL contribution to calculate QALY	0.771–0.835 (ablation) 0.769–0.824 (AAD)	0.673–0.788 (ablation) 0.664–0.769 (AAD)	£5657 (€6335)	0.097	£58 208 (€65 193)	RCT
Mortality probability at baseline	0.814% (ablation)−0.839% (AAD)	0.6% and 1.5%	£5492 (€6151) and £6001 (€6721)	0.026 and 0.009	£206 689 (€231 492) and £657 342 (€736 223)	Registry
Difference in mortality probability	0.025%	0.239%	£5798 (€6494)	0.202	£28 631 (€32 067)	Expert opinion
Mortality probability incremental rise per year	0.1%	0.3%	£5782 (€6476)	0.033	£175 290 (€196 325)	Expert opinion
Operative mortality of ablation	0.5%	0.25% and 3%	£5661 (€6340) and £5604 (€6276)	0.046 and −0.078	£123 562 (€138 389) and AAD dominates	Registry
Baseline readmission probability per month	1.332% (ablation) −1.666% (AAD)	0.23%–2.273%	£3204 (€3588)	0.052	£61 254 (€68 604)	RCT*
Repeat ablation probability per month	19% and 24%	10% and 33%	£5893 (€6600) and £5339 (€5980)	0.039 and 0.039	£151 895 (€170 122) and £133 588 (€149 619)	Expert opinion
Adverse event probability for ablation	3%	6.5%	£5657 (€6335)	0.039	£144 093 (€161 384)	Registry
Adverse event probability of AAD per month	1.279%	3.75%	£5657 (€6335)	0.042	£134 711 (€150 876)	Registry
Rate of amiodarone use in ablation group	20%	10% and 80%	£5454 and £6879	0.038 and 0.037	£141 713 (€158 719) and £182 362 (€204 245)	Registry

*Range of readmission probability selected from VTACH and SMS-VT to derive the largest difference in readmission between two treatments. Additionally, please see online supplementary appendix A for a two-way sensitivity analysis altering disutility.

AAD, antiarrhythmic drug; EQ-5D, EuroQol-5 Dimension; HRQL, health-related quality of life; ICER, incremental cost-effectiveness ratio; QALY, quality-adjusted life-year; RCT, randomised clinical trial; SMS, Substrate Modification Study; VT, ventricular tachycardia.

**Figure 2:**
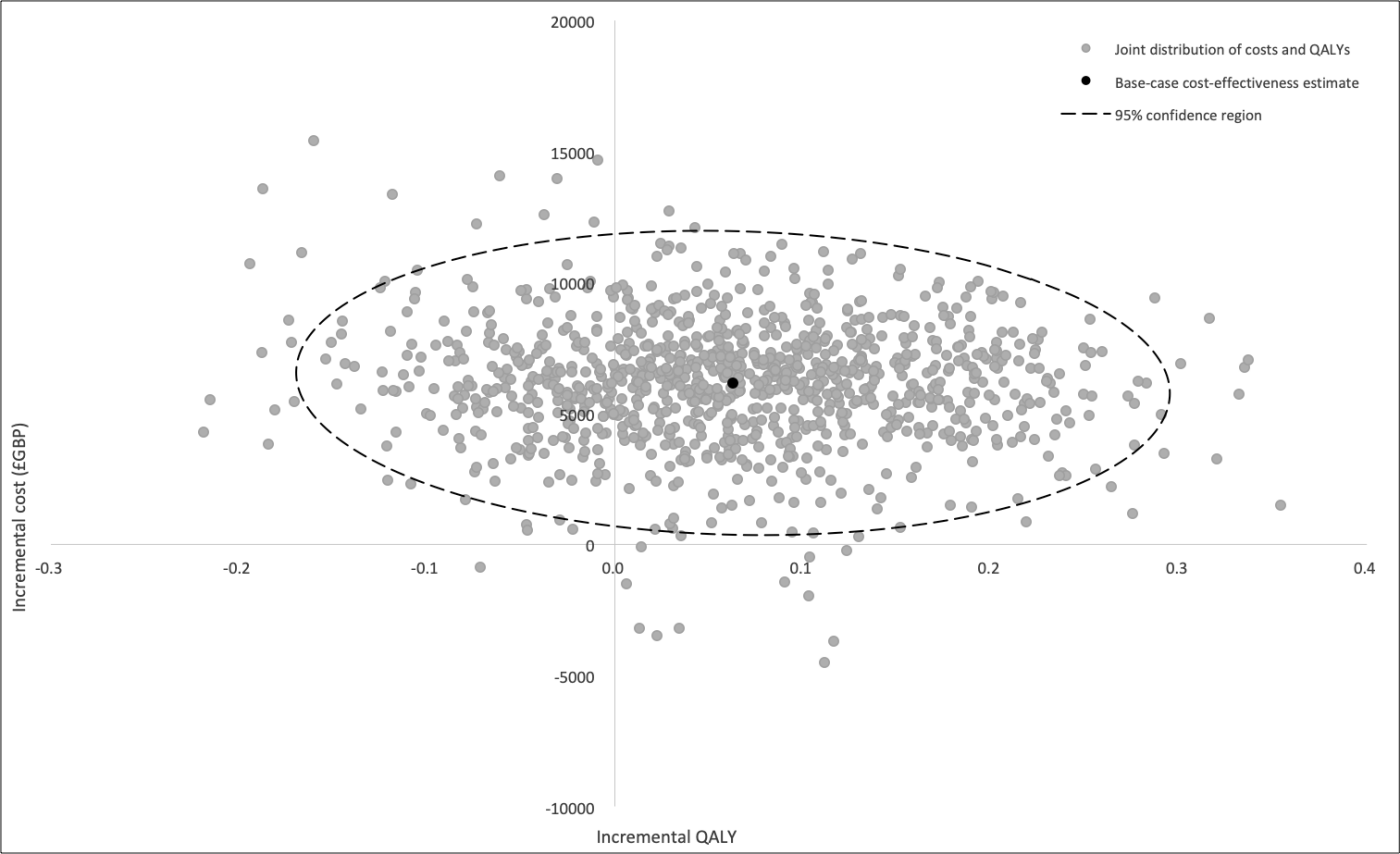
Cost-effectiveness plane demonstrating variation in results depending on probabilistic sensitivity analysis, with 95% of results within dotted line. The plane illustrates that the distribution of costs and QALYs lies mostly in the North-East and North-West quadrants. This means that while ventricular tachycardia (VT) ablation appears to be more costly there is more uncertainty about its effectiveness (95% confidence region crosses zero). QALY, quality-adjusted life-year.

**Figure 3:**
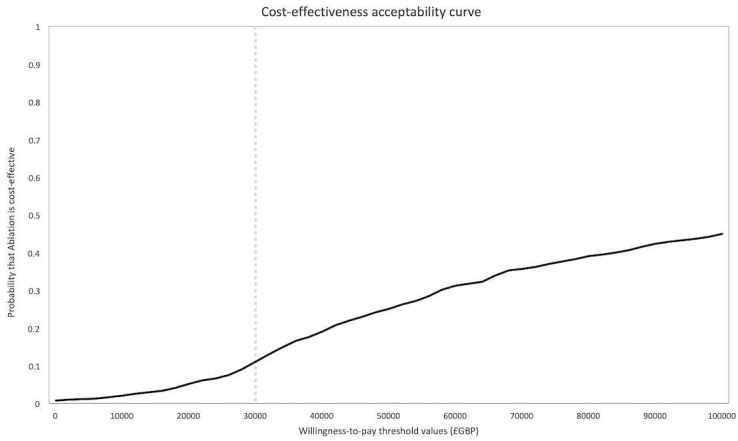
Cost-effectiveness acceptability curve for catheter ablation of ventricular tachycardia (VT) compared with antiarrhythmic drug (AAD) therapy in patients with ischaemic cardiomyopathy and an implantable cardioverter-defibrillator (ICD).

Results of the probabilistic sensitivity analysis are reported in the cost-effectiveness plane (figure 2)—this is generated through running the model 1000 times. Figure 3 reports the probability of VT ablation being cost-effective compared with AAD across a wide range of WTP thresholds (cost-effectiveness acceptability curve). The overall probability of catheter ablation being cost-effective is about 11%, at the WTP threshold of £30 000 per QALY.

## Discussion

The overall benefit of any intervention has to be seen in the context of the patient’s health status. In practice, VT ablation is a specialist procedure mainly used as secondary prevention to reduce shocks and hospitalisations, often in those with the most frequent or sustained episodes of VT. It can be highly effective in a proportion of these patients, however, the underlying morbidity and burden of their cardiovascular disease can mean deterioration independent of the VT and/or ablation procedure.^19^

Given that ablation confers benefit to patients through reductions in VT events and not through lowering mortality, from a health economist’s perspective, assessing whether it is cost-effective will be determined by the cost and HRQL measurement. When the HRQL difference is small between ablation and AAD, this has the effect of making the ICER large.

It is important to highlight that although cost-effectiveness is a measure of whether an intervention is efficient for the system, this is not the only determinant of system-level decision-making. For example, VT ablation is unlikely to be affected by the budget constraints of most countries who use this technology, given the relative infrequency to which it is performed. One must also note that regardless of whether VT ablation is an efficient use of resources or not, for some patients, it may be their only treatment option available, affording them a chance of relief from symptoms which is of immeasurable benefit to them.

However, by studying catheter ablation of VT as an exemplar, this paper highlights some significant deficiencies in the current trial evidence base where application of health economic analyses could go awry if policymakers conduct them with limited data and a priori agendas.

Currently, patient-reported HRQL in VT ablation trials is insufficient to draw robust conclusions regarding cost-effectiveness, with an additional contention that of the HRQL data that have been collected, this was measured too infrequently to have captured all the differences in health status between the two interventions. This has the potential of underestimating the real benefits of the procedure—both to patients as well as to the system in terms of its cost-effectiveness. In health conditions where short-term penalties to utility may be frequent—such as admissions with VT—the assumption of accurately capturing all these by only asking patients once or twice a year is debatable and recall bias is an important confounder.

This paper is the first in the area of VT to use all available RCT-level evidence to inform the economic model inputs. Additionally, a ‘real-world’ micro costing method ensured that the reported additional costs of VT ablation were as reflective of current practice as possible. This same approach was taken in choosing the health states of the model, which sought to reflect as closely as possible the natural progression of the disease and the clinical context as well as being pragmatic in light of available data from patient-reported HRQL.

### ​Challenges and limitations in performing model analysis

Any model is only as good as the set of its inputs. Like others, limitations will be determined by the quality of evidence used to inform the model design. The analysis deliberately omitted one of the main clinical differences borne out of the RCTs between AAD and ablation—the number of ICD shocks or time to first VT recurrence. The principal reason for this was a lack of reliable HRQL data that accurately captured the disutility of experiencing a shock or VT recurrence at a specific moment in time. There is also the consideration that some VT recurrences (particularly if slow) could have caused little in the way of symptoms and/or have been treated with antitachycardia pacing alone. We therefore elected only to model VT storm—this was taken to put the patient into the readmission health state of our model. Thus, rather than relying on significant use of expert opinion to infer utilities performed in previous studies,^10 14^ we included only health states with patient-reported utilities supported by verifiable objective data. This is an important distinction as the objectivity of expert opinion by interested parties is difficult to confirm.

However, even with the available RCT data, it is not entirely possible to distinguish whether hospitalisations or patient-reported HRQL differences could be attributable solely to VT recurrences, or to other cardiovascular or non-cardiovascular causes.

Additionally, the RCTs had differences in their comparators, for example, VANISH compared escalated AAD treatment with ablation. It was not possible for our model to examine differences in subgroups such as this. As a work around, one of the sensitivity analyses conducted examined the impact on the ICER if VT ablation had an even larger effect in reducing readmissions—a cohort of patients who by definition would be deriving the most benefit and may reflect the escalated AAD treatment group who undergo ablation. In this scenario, there was a significant drop in the ICER to £61 738 (€69 147)—and with the addition of heavier weighting of disutility of readmission, this further trended towards the UK’s WTP of £30 000 (see online supplementary appendix A).

We used the SF-36 instrument as the source of HRQL data to calculate the QALYs—this was in part so that we could pool all of the available RCT-level HRQL outcomes. Contrastingly, a recent cost-effectiveness study using EQ-5D from the VANISH trial concluded catheter ablation of VT was cost-effective.^11^ However their results are based on a single within-trial analysis over a shorter time frame. Of note, both SF-36 and EQ-5D are not disease specific and there is evidence of divergent quality of life results depending on which scale is used.^20^

Beyond the RCT follow-up period, reference to large VT ablation registries with long-term follow-up, as well as cohort data from our own institution was used to guide parameter changes.^16 17^ These additional assumptions had the smallest effect on the base-case analysis due to its shorter time horizon. We did not perform a systematic review of the literature to identify all non-RCT studies to guide selection of missing model input parameters for the principal reason that there are few comparable studies in this specific cohort of patients. Of existing studies, a heterogeneous group of patients with different characteristics, for example, heart failure without ICD, could report significantly different utilities impacted by differences in their baseline burden of disease.

It is also important to note that costs can vary significantly depending on the country and setting, and for those that use a WTP, there are significant differences in the societal opinion on what the value of this should be.^21^

Overall, the conclusions from our model can be changed by relatively small differences in calculated QALYs associated with either ablation or AAD treatment. Future trials should place greater emphasis on the collection of HRQL data. Indeed, this echoes the message from the European Society of Cardiology in 2014, which set forth a call for their mandatory integration into all future trials.^22^ As the cost of healthcare continues to rise, it is important that accurate measurement and reporting of the cost-effectiveness of different treatments occurs, irrespective of how funding decisions are made in different countries and healthcare systems. The role of more widespread patient-reported HRQL—or patient-reported outcome measures in general—is vital. More frequent sampling use of a standardised HRQL measuring score could have significant policy implications in this area and beyond.

## Conclusion

This is the first study to examine the RCT evidence base of catheter ablation of VT, from the perspective of the quality of their patient-reported HRQL and subsequent cost-effectiveness calculation. It is striking that given discussion of quality of life in patients with ICDs is a class I recommendation^8^ from international guidelines, there are still considerable inaccuracies and knowledge gaps in the reporting of HRQL. If both trial and observational data included more frequent and standardised use of HRQL data, more robust economic analyses and firmer conclusions about cost-effectiveness will be drawn.
