# Supplementary material for: Cost-effectiveness of ablation of ventricular tachycardia in ischaemic cardiomyopathy: limitations in the trial evidence base
Source: Open Heart. 2020 Jan 28;7(1):e001155. doi: 10.1136/openhrt-2019-001155 (PMC6999675; doi:10.1136/openhrt-2019-001155)
Supplement: Supplementary data [file openhrt-2019-001155supp001.pdf]

Appendix A

Equations used to inform transition probabilities:

Ablation arm

For cycle 1:
[H7] Success =G6*(1-omrPABL)+H6*(1-rrr-mr)+I6*(1-(2.5*mr))*0.81+J6*(1-omrRABL-mr-rrr)
[I7] Readmission =H6*rrr+J6*rrr
[J7] Reablation =I6*(1-(2.5*mr))*0.19
[K7] Death =H6*mr+(I6)*(2.5*mr)+J6*(omrRABL+mr)+G6*omrPABL+K6
[L7] Check = =SUM(H7:K7)

AAD arm

For cycle 1:
[H7] AAD maintenance =G6+H6*(1-rrrr-mr)+I6*(1-mr)*0.76+J6*(1-omrRABL-mr-rrrr)
[I7] Readmission =H6*rrrr+J6*rrrr
[J7] Switch to ablation =I6*(1-mr)*0.24
[K7] Death =H6*mr+I6*mr+J6*(omrRABL+mr)+G6*omrAADi+K6
[L7] Check = =SUM(H7:K7)

Key

G6 – cell in spreadsheet representing start of simulation with 1000 patients
H7 – cell in spreadsheet representing number of patients in success state
I7– cell in spreadsheet representing number of patients in readmission state
J7 – cell in spreadsheet representing number of patients in reablation state
K7 – cell in spreadsheet representing number of patients in death state
L7 – cell in spreadsheet that checks to ensure all patients are accounted for

*For each subsequent row, the next cycle will have different numbers, though the K column is additive as it represents a terminal inescapable state.
**For the L column, this number always totaled 1000, an important step in ensuring the internal validity of the model.

omrPABL – procedural mortality following primary ablation
omrRABL – procedural mortality following repeat ablation
omrAADi – mortality assigned to AAD strategy
rrr – readmission risk for ablation strategy
rrrr – readmission risk for AAD strategy
mr –mortality rate (altered incrementally by 0.1% after every 12 cycles)

Transition probability of 19% chance from readmission to reablation in Ablation group and 25% chance in AAD group (see appendix B)

Model inputs based on RCTs – used to apportion weighted rates depending on sample size. For the VISTA trial, both treatment arms' were used as part of calculating ablation input rates for the model.

Confidential: For Review Only

Chen Y, *et al. Open Heart* 2020; 7:e001155. doi: 10.1136/openhrt-2019-001155

Two-way sensitivity analysis

If the disutility experienced by a readmission is adjusted to model for a high short-term penalty (0.004 utility over 1 month cycle), then in combination with a large difference in readmission, the ICER approaches the UK's WTP:

Strategy	Mean total cost	Mean total QALYs	Incremental cost	Incremental QALY	ICER
Ablation	£8,964 (€10,039)	2.805	£3,204 (€3,555)	0.104	£30,684 (€34,366)
AAD	£5,760 (€6,451)	2.701			

The assumption is reasonable given that annual utility of 0.781 at baseline would be shifted to 0.716 after 1 admission and 0.6515 after two, at the end of 12 cycles (or 1 year).

Confidential: For Review Only

Appendix B

Further sources of information and assumptions used in Markov model

1a. Monetary costs

https://improvement.nhs.uk/resources/reference-costs/
Joint Formulary Committee (2018) *British National Formulary 75 March-September 2018.*

1b. Cost of length of stay for readmission

Predicated on average length of stay for readmission (4 days)

Viles-Gonzalez JF, Arora S, Deshmukh A, Atti V, Agnihotri K, Patel N et al. Outcomes of Patients Admitted with Ventricular Arrhythmias and Sudden Cardiac Death in the United States, Heart Rhythm (2018), doi: https://doi.org/10.1016/j.hrthm.2018.09.007. *Supplementary material*

2. Readmission associated with x2.5 increased mortality in that cycle.

Duray GZ, Schmitt J, Richter S, Israel CW, Hohnloser SH. Arrhythmic death in implantable cardioverter defibrillator patients: a long-term study over a 10 year implantation period. Europace (2009) 11, 1462–1468

3. Disutility of readmission -0.02 and repeat procedure -0.04, and reablation with adverse event

Sanders GD, Hlatky MA, Owens DK. Cost-effectiveness of implantable cardioverter-defibrillators. N Engl J Med 2005;353:1471–80.

Neyt M, Van Brabandt H, Devos C**.** The cost-utility of catheter ablation of atrial fibrillation: a systematic review and critical appraisal of economic evaluations. *BMC Cardiovascular Disorders* 2013**13**:78

Swinburn P, Shingler S, Ong SH, Lecomte P, Lloyd A. Assessing the health-related quality of life in patients hospitalised for acute heart failure. Br J Cardiol 2013;20:72–6.

Lewis EF, Li Y, Pfeffer MA, Solomon SD, Weinfurt KP, Velazquez EJ et al. Impact of cardiovascular events on change in quality of life and utilities in patients after myocardial infarction: a VALIANT study (valsartan in acute myocardial infarction). J Am Coll Cardiol HF 2014;2:159–65.

4. Probability of transition from repeat admission to reablation on that cycle

Sionitis KC, Kim HM, Stevenson WG, Fujii A, Bella PD, Vergara P et al. Prognostic Impact of the Timing of Recurrence of Infarct-Related Ventricular Tachycardia After Catheter Ablation. Circ Arrhythm Electrophysiol. 2016 December ; 9(12):

5. Cost of readmission – assuming mean length of stay is 4 days

Viles-Gonzalez JF, Arora S, Deshmukh A, Atti V, Agnihotri K, Patel N et al. Outcomes of Patients Admitted with Ventricular Arrhythmias and Sudden Cardiac Death in the United States. Heart Rhythm. 2018 Sep 17. pii: S1547-5271(18)30919-6

6. Increased mortality of 2.5 Hazard ratio for those in readmission state, for that cycle

Sanders P, Connolly A, Nabutovsky Y, Fischer A, Saeed M. Increased Hospitalizations and Overall Healthcare Utilization in Patients Receiving Implantable Cardioverter-Defibrillator Shocks Compared With Antitachycardia Pacing. JACC Clinical Electrophysiology 2018 Feb;4(2):243-253

7. Method used to assume standard error in Probabilistic sensitivity analysis

Buxton M, Caine N, Chase D, Connelly D, Grace A, Jackson C et al. A review of the evidence on the effects and costs of implantable cardioverter defibrillator therapy in different patient groups, and the modelling of cost-effectiveness and cost-utility for these groups in a UK context. Health Technol Assess 2006;10(27):1-180.

Basis of parameters one-way sensitivity analysis used in Markov model

1. Major adverse event rate up to 6.5% for catheter ablation

Confidential: For Review Only

Katz DF, Turakhia MP, Sauer WH, Tzou WS, Heath RR, Zipse MM et al. Safety of Ventricular Tachycardia Ablation in Clinical Practice. Circ Arrhythm Electrophysiol. 2015;8:362-370.

2. Baseline death rate of 0.6% and 1.5%

Bunch TJ, Weiss JP, Crandall BG, Day JD, May HT, Bair TL et al. Patients treated with catheter ablation for ventricular tachycardia after an ICD shock have lower long-term rates of death and heart failure hospitalization than do patients treated with medical management only. Heart Rhythm. 2014 Apr;11(4):533-40.

Tzou WS, Tung R, Frankel DS, Vaseghi M, Bunch TJ, Di Biase L et al. Ventricular Tachycardia Ablation in Severe Heart Failure An International Ventricular Tachycardia Ablation Center Collaboration Analysis. Circ Arrhythm Electrophysiol. 2017;10:e004494.

3. Rate of amiodarone use up to 80%

Tzou WS, Tung R, Frankel DS, Vaseghi M, Bunch TJ, Di Biase L et al. Ventricular Tachycardia Ablation in Severe Heart Failure An International Ventricular Tachycardia Ablation Center Collaboration Analysis. Circ Arrhythm Electrophysiol. 2017;10:e004494.

4. Adverse event rate of AAD up to 3.75%

Kowey PR, Crijns HJ, Aliot EM, Capucci A, Kulakowski P, Radzik D et al. Efficacy and Safety of Celivarone, With Amiodarone as Calibrator, in Patients With an Implantable Cardioverter-Defibrillator for Prevention of Implantable Cardioverter-Defibrillator Interventions or Death The ALPHEE Study. Circulation. 2011;124:2649-2660.

5. Procedural mortality of 3%

Vakil K, Garcia S, Tung R, Vaseghi M, Tedro U, Della Bella P et al. Ventricular Tachycardia Ablation in the Elderly An International Ventricular Tachycardia Center Collaborative Group Analysis. Circ Arrhythm Electrophysiol. 2017;10:e005332.

Confidential: For Review Only

Chen Y, *et al. Open Heart* 2020; 7:e001155. doi: 10.1136/openhrt-2019-001155
